# The Promise of Neuroimmune Targets for Treating Drug Addiction and Other Psychiatric Disorders: Granulocyte-Colony Stimulating Factor Exemplification

**DOI:** 10.3389/fpsyt.2020.00220

**Published:** 2020-03-17

**Authors:** Mehdi Farokhnia, Anthony L. Berger, Hollis C. Karoly, Lara S. Hwa, Florence P. Varodayan

**Affiliations:** ^1^Clinical Psychoneuroendocrinology and Neuropsychopharmacology Section, National Institute on Drug Abuse Intramural Research Program and National Institute on Alcohol Abuse and Alcoholism Division of Intramural Clinical and Biological Research, National Institutes of Health, Bethesda, MD, United States; ^2^Center on Compulsive Behaviors, National Institutes of Health, Bethesda, MD, United States; ^3^Johns Hopkins Bloomberg School of Public Health, Johns Hopkins University, Baltimore, MD, United States; ^4^Department of Neurology, University of California San Francisco, San Francisco, CA, United States; ^5^Institute for Cognitive Science, University of Colorado Boulder, Boulder, CO, United States; ^6^Bowles Center for Alcohol Studies, University of North Carolina School of Medicine, Chapel Hill, NC, United States; ^7^Behavioral Neuroscience Program, Department of Psychology, Binghamton University-SUNY, Binghamton, NY, United States

**Keywords:** addiction, immune system, neuroimmune, granulocyte-colony stimulating factor, medication development

Drug addiction is a chronic relapsing disease leading to allostatic alterations in the brain’s reward, stress, and executive function systems (1). The etiology and pathophysiology of addiction are complex, with multiple genetic and environmental factors involved. Despite considerable progress in understanding the neurobiology of substance use disorders (SUDs), pharmacological treatments remain limited in number and efficacy. Directly targeting dysregulated neurotransmitter systems to treat SUDs can be problematic and may cause undesirable side effects. Thus, there is growing interest in investigating the role of peripheral/modulatory pathways (e.g., immune factors, hormones, gut microbiome) in addiction, with the ultimate goal of identifying novel therapeutic targets (2–4).

Bidirectional interactions between the nervous system and immune system, collectively known as the “neuroimmune system”, regulate a wide range of physiological and pathological processes (5). While several studies have linked general neuroinflammation and neuropsychiatric disorders [e.g., depression (6), schizophrenia (7), addiction (8, 9)], only recently have specific immune factors been shown to modulate neuronal activity and complex behavioral processes (10–12). Alcohol and other drugs of abuse stimulate the neuroimmune system, primarily by activating microglial cells and inducing immune-related gene expression. These neuroimmune responses can produce lasting changes in neuronal structure and function, including cellular damage, synaptic remodeling, and altered neurotransmission (e.g. dopaminergic and glutamatergic signaling). There is also growing evidence indicating that microglial activation and unbalanced production of cytokines, chemokines, and reactive oxygen species can play causal roles in the development and progression of SUDs (13, 14).

Granulocyte-colony stimulating factor (G-CSF) is a ~20-kDa protein produced by bone marrow stromal cells, endothelial cells, macrophages, and fibroblasts, as a peripheral hematopoietic growth factor (15). Both G-CSF and its receptor (G-CSF-R) are also present in the brain (expressed in neurons and glia), and G-CSF signaling has been shown to modulate a variety of neuronal functions (16). G-CSF has potent anti-inflammatory properties and inhibits the production of pro-inflammatory cytokines [e.g., tumor-necrosis factor alpha (TNF-α), interleukin-1 and interferon gamma] (17). It is also an essential neurotrophic/neuroprotective factor that inhibits apoptosis, stimulates neurogenesis, recruits neutrophils for neural tissue repair and to fight infection, and mobilizes stem cells to injured brain areas to increase neuroplasticity (18, 19). Given these important regulatory effects, G-CSF has been proposed as a potential treatment for conditions associated with aberrant neural function, including neuropsychiatric disorders.

In a series of rodent experiments, Kutlu et al. (20) investigated the effects of G-CSF administration on different measures of motivation, cognition, dopaminergic neurotransmission, and gene expression. The researchers first made use of a sucrose threshold task, in which the number of lever presses necessary to earn a reward was progressively increased. Animals treated with G-CSF pressed more for sucrose than those treated with vehicle, indicating enhanced motivation. Next, a reversal learning task was employed, where the previously inactive lever became the active lever, forcing the animals to alter their responses in order to obtain a reward. Animals treated with repeated G-CSF injections learned the new response-contingency faster and made fewer incorrect responses than their vehicle-treated counterparts, suggesting enhanced cognitive function. Of note, animals previously treated with G-CSF failed to show enhanced learning when the contingencies were reversed a second time, suggesting that the enhanced cognitive function required G-CSF to be on board.

To evaluate the biological underpinnings of these observations, the researchers next turned to *ex vivo* fast-scan cyclic voltammetry to assess dopaminergic function in the nucleus accumbens (NAc)—a region heavily implicated in reward processing. Repeated systemic administration of G-CSF increased electrically-evoked dopamine release in the NAc, without altering dopamine uptake or transport. Acute application of G-CSF onto the brain slice failed to alter NAc dopamine transmission, indicating that G-CSF does not act directly at dopaminergic terminals in the NAc, but perhaps on their cell bodies in the ventral tegmental area (VTA) or other converging circuits [e.g., input from the medial prefrontal cortex (mPFC)]. Finally, quantitative polymerase chain reaction revealed that repeated G-CSF treatment suppressed pro-inflammatory cascades in the NAc, as demonstrated by reduced TNF-α gene expression, while acute G-CSF increased the expression of several dopaminergic genes (e.g., dopamine receptors D1 and D2) (20). Collectively, these results indicate that immune mediators, such as G-CSF, can exert powerful and dynamic effects on both behavioral and biological measures of reward, suggesting that the neuroimmune system may be a viable target for the treatment of drug addiction and other psychiatric disorders.

A previous study by the same group delineated the link between G-CSF and the biobehavioral response to cocaine. Specifically, Calipari et al. (21) found that serum G-CSF concentrations were significantly increased following volitional and non-volitional cocaine exposure, and these increases were positively correlated with the extent of cocaine sensitization and self-administration. Consistent with these peripheral changes, G-CSF and G-CSF-R genes expression in the NAc were also increased following acute and repeated cocaine injections. These findings provided preliminary evidence that exposure to cocaine is associated with changes in the endogenous G-CSF system. Next, in order to test causality, the researchers examined the effects of exogenous G-CSF administration on different measures of cocaine seeking and consumption. An acute dose of experimenter-administered G-CSF potentiated cocaine-induced neuronal activation in the mPFC and NAc, though G-CSF alone (in the absence of cocaine) did not alter neuronal activity. Systemic injection of G-CSF also enhanced cocaine-induced locomotor sensitization, conditioned place preference for cocaine, motivation for cocaine, and cocaine intake, with no effect on motivation for food reward or sucrose preference. Notably, the latter observation contrasts with the report mentioned before (20), where G-CSF enhanced sucrose motivation and consumption. This discrepancy may be related to the different experimental designs (i.e., sucrose threshold task versus two-bottle sucrose preference task), animals’ motivational states (i.e. food deprived versus non-deprived), and/or other unknown factors. Procedures requiring more effort (e.g., operant self-administration) or the engagement of differing motivational states may recruit specific circuitries in which G-CSF exerts a stronger modulatory role.

Future work is warranted to determine the extent to which G-CSF influences behavioral correlates of drug and natural rewards, as well as its regulatory effects in other addiction-related brain regions. As a first pass, Mervosh et al. (22) used an unbiased quantitative approach to examine proteomic changes in the VTA induced by G-CSF alone and its co-administration with cocaine. Ingenuity Pathway Analysis (IPA) revealed that the proteins influenced by G-CSF were also regulated by Fragile X mental retardation protein (FMRP) and mammalian target of rapamycin (mTOR), both key regulators of synaptic plasticity and intricately involved in SUDs (23, 24). These findings further demonstrate that neuroimmune factors like G-CSF can have profound and multifaceted effects on the brain, underscoring the need to tease apart their region- and circuit-specific actions in relation to drug seeking behaviors.

As a whole, the aforementioned data elucidate novel mechanisms whereby peripheral administration of an anti-inflammatory cytokine (G-CSF) enhances the activity of a central reward circuit (mesolimbic dopamine system) and modulates both motivational and cognitive aspects of reward processing. The sucrose threshold task used in this study should be highlighted as this behavioral economics self-administration protocol allows for the dissociation of A) the animal’s preferred level of consumption when effort is low (i.e. preferred intake level) and B) the maximal effort the animal will expend to maintain its preferred intake level (i.e. motivation to consume). The researchers determined that G-CSF increased both sucrose intake and motivation. As demonstrated by the reversal learning task, cognitive flexibility was also enhanced by repeated, but not acute, G-CSF injections. This is an important finding given that cognitive deficits play substantial roles in many neuropsychiatric diseases, including addiction, and most of the currently available pharmacotherapies do not address these impairments (25). A single dose of G-CSF (50 μg/kg) was used in these experiments, and acute versus repeated treatment generated different results. Therefore, follow-up studies on G-CSF’s time course of action and ideal dosage, as well as its effects on other measures of reward and cognition are crucial future steps. From a mechanistic perspective, NAc dopaminergic signaling was characterized as a key target for G-CSF’s central functions. Future studies should investigate G-CSF’s effects on other brain regions (e.g. PFC, amygdala, hippocampus) and neurotransmitter systems (e.g. glutamate, GABA, norepinephrine, serotonin) implicated in the pathophysiology of addiction. Future research should also delve into the interplay between G-CSF and stress systems, as neuroimmune mechanisms are closely linked to the neurobiology of stress (26), and chronic stress is involved in the development and progression of several neuropsychiatric disorders (27).

Medication development for neuropsychiatric disorders, especially addiction, is a complex, lengthy, and expensive process with low success rates. Therefore, the notion that G-CSF may represent an effective therapeutic target is of particular relevance and interest. Recombinant G-CSF is already approved by the FDA and has been tested in clinical trials for other indications—an advantage which may facilitate translation of the present preclinical findings into human research. As an initial bench-to-bedside step, proof-of-concept human laboratory studies may provide valuable information on the effects of G-CSF administration in patients with SUDs. As demonstrated by Calipari et al. (21), G-CSF does not appear to have rewarding properties on its own nor does it alter baseline metabolism, which are favorable characteristics from a medication development standpoint. Administration of recombinant G-CSF in humans is generally safe and well-tolerated; the most common side effects, which are usually mild-to-moderate and self-limiting, include headache, fatigue, myalgia, bone pain, nausea, and vomiting (28, 29). Since G-CSF is an immunomodulatory agent, it could also improve psychiatric (e.g. depression, anxiety) and medical (e.g. HIV infection, liver disease) comorbidities of addiction, though this hypothesis requires investigation. Possible sex differences in response to G-CSF is another consideration, especially because biobehavioral correlates of the neuroimmune system are different between males and females (30). Finally, while additional preclinical experiments with G-CSF are needed (e.g., other drugs of abuse, animal models of dependence), complementary pharmacological manipulations (e.g., G-CSF neutralizing antibodies, G-CSF-R agonists/antagonists) may also provide a deeper insight into the applicability of this pathway as a viable target for treating drug addiction and other psychiatric disorders.

## Author Contributions

MF and FV were responsible for the conceptualization of this paper. MF, AB, HK, LH, and FV developed the rationale, wrote the first draft, critically reviewed the content, and approved the final manuscript for publication.

## Funding

This work was supported by NIH intramural funding ZIA-AA000218 (Section on Clinical Psychoneuroendocrinology and Neuropsychopharmacology), jointly supported by the NIDA Intramural Research Program and NIAAA Division of Intramural Clinical and Biological Research (MF); NIH Center on Compulsive Behaviors, funded by the NIH Deputy Director for Intramural Research (DDIR) Innovation Award (MF); NIAAA K99 AA027576 grant (LH); NIAAA K99 AA025408 grant (FV); and Developmental Exposure Alcohol Research Center (DEARC) at Binghamton University, P50 grant AA017823 (FV). The content of this article is solely the responsibility of the authors and does not necessarily represent the official views of the funders, which had no role in the development of this article.

## Conflict of Interest

The authors declare that the research was conducted in the absence of any commercial or financial relationships that could be construed as a potential conflict of interest.
